# QTL mapping of flowering time in *Brassica napus*: a study on the interplay between temperature and day length after vernalization

**DOI:** 10.3389/fpls.2025.1513353

**Published:** 2025-05-09

**Authors:** Eva Heinrich, Antje Schierholt, Christian Möllers

**Affiliations:** ^1^ Department of Crop Sciences, Division of Crop Plant Genetics, Georg-August-University Göttingen, Göttingen, Germany; ^2^ Department of Crop Sciences, Division of Plant Breeding Methodology, Georg-August University Göttingen, Göttingen, Germany

**Keywords:** epistasis, genetic variation, circadian rhythm, photoperiod, gene homology, winter rape, epigenetic, vernalization

## Abstract

Flowering is a critical life stage for plants, and the regulation of flowering is heavily influenced by environmental factors and is genetically very complex. In oilseed rape (*Brassica napus* L.), a major oil crop, yield is heavily dependent on successful flowering. Until now, the influences of day length and temperature on flowering time have mostly been studied in spring-type rape, although they also affect flowering in winter oilseed rape after vernalization, and changing climate conditions alter springtime temperatures. In this study, a doubled haploid population derived from a cross between a winter and a spring-type oilseed rape was examined for the effect of cool and warm temperatures (11°C and 22°C) in combination with long and short days (8/16-h light) on flowering time after vernalization. Quantitative trait locus (QTL) analysis revealed major QTLs for flowering time in two homologous regions on chromosomes C06 and A07, which were found to interact epistatically. It was found that temperature can either delay or promote flowering depending on day length and genotype, highlighting the complex interplay between these factors. Our study provides new insights into the genetic basis of flowering time regulation in *B. napus*, especially after vernalization, and highlights the importance of considering the interplay between temperature and day length in breeding programs for this crop, particularly in the context of climate change.

## 1 Introduction

Flowering time is regulated in a complex network with different pathways that interact with each other and are well studied in *Arabidopsis thaliana* (Blümel et al., 2015; Quiroz et al., 2021). The internal and external signals are thereby controlling the autonomous and gibberellin pathways, as well as the vernalization, temperature, and day length pathways. Most of the environmental cues are sensed in the leaves and lead to the expression of *FLOWERING LOCUS T* (*FT*) through signaling cascades. The FT protein travels to the apical meristem and initiates the generative phase (Jaeger et al., 2013). Oilseed rape (*Brassica napus* L.) is closely related to *Arabidopsis*. However, a genome triplication occurred in the evolution of the genus *Brassica*, and the hybridization of *Brassica rapa* and *Brassica oleracea* led to the allopolyploid species *B. napus* with the A and C subgenomes (Chalhoub et al., 2014). Therefore, genes occur with a high copy number variation, including flowering time genes, which makes flowering time regulation in the *B. napus* complex (Schiessl et al., 2017; Schiessl, 2020).

Vernalization is the initiation of flowering through a prolonged cold period. In crop plants, the requirement of a vernalization period to initiate flowering separates winter crops from spring crops; therefore, the vernalization pathway is well studied in *B. napus* (Ferreira et al., 1995; Raman et al., 2016; Richter and Möllers, 2018; Schiessl et al., 2019).

Studies in *Arabidopsis* have shown the complexity of the molecular mechanisms for the regulatory pathways of day length and temperature. The influence of day length is often regulated over the inner circadian clock with the central regulator CONSTANS, a direct FT activator (Takagi et al., 2023). They are known to interact with each other, as well as with the plant age and the gibberellin pathway, making this one of the most complex pathways for flowering (Song et al., 2013; Kim and Sung, 2014; Blümel et al., 2015). Even though this is the case, many studies on the influence of temperature on flowering have been done without considering other abiotic factors like day length (Schiessl, 2020; Jiang, 2023). In particular, the effect of temperature and day length after vernalization is not studied in *B. napus*. Like *Arabidopsis* (Amasino and Michaels, 2010), oilseed rape is a long-day plant, for which longer day length and higher temperature generally lead to earlier flowering (Major, 1980; King and Kondra, 1986; Mendham and Salisbury, 1995; Nanda et al., 1996; Robertson et al., 2002; Nelson et al., 2014). So far, quantitative trait locus (QTL) mapping studies identified chromosomes A02, A03, A10, C03, C04, C05, and C09 as carrying photoperiod-sensitive genes (Robert et al., 1998; Axelsson et al., 2001; Cai et al., 2008; Luo et al., 2014; Rahman et al., 2018). However, most of the molecular markers used at that time do not allow identification of their physical positions on current reference genomes (Chalhoub et al., 2014; Sun et al., 2017; Lee et al., 2020).

For the effect of temperature, only very few studies have been done, all of them on spring-type rapeseed. Abelenda et al. (2023) studied the difference in flowering time between 21°C and 28°C in different spring-type cultivars. Most cultivars delayed flowering time under the higher temperature, but one genotype accelerated flowering and showed different *FT* expression. Salisbury and Green (1991) reported interactions between temperature and day length on flowering time in spring genotypes of European, Canadian, and Australian origins. In a Canadian spring oilseed rape doubled haploid (DH) population, Rahman et al. (2018) detected QTLs for flowering time for different day lengths and temperature regimes. In *B. rapa*, Xiao et al. (2019) mapped flowering time QTLs for responses to ambient temperature and photoperiod on nearly all chromosomes.

In conclusion, all these studies have shown genotypic differences in response to day length and temperature regarding flowering time regulation. However, the majority of those studies in *B. napus* were done on spring types or the effect of different temperatures during or before vernalization in winter types. Even though rising temperatures during winter and early spring caused by climate change are evident in areas of winter oilseed rape cultivation, the reaction of flowering time in winter oilseed rape to temperature and its interaction with day length has been understudied. Therefore, the objectives of the present work were to test the impact of day length and temperature on flowering time in fully vernalized plants of the DH population DH4079 × Express617 and to assess the interaction between temperature and day length. To achieve these objectives, plants vernalized for 9 weeks were grown under four different controlled conditions with combinations of short and long days (8 and 16 h) and at two temperature regimes (11°C and 22°C) to determine days to flowering. A single-nucleotide polymorphism (SNP) marker-based linkage map was used to map QTLs and identify candidate genes.

## 2 Methods

### 2.1 Plant material

The inbred line 617 from the winter oilseed rape cultivar Express (Norddeutsche Pflanzenzucht Hans-Georg Lembke KG, Holtsee, Germany) and the doubled haploid line DH4079 (Ferrie, 2003) from the Swedish spring-type cultivar Topas were crossed to generate F1 seeds. A DH population consisting of 184 lines was developed from clonally propagated F1 plants as described by Valdés et al. (2018).

### 2.2 Day length and temperature experiment

The effect of day length and temperature on the flowering time of fully vernalized plants was determined in a split–split plot design with two-factor levels in temperature (11°C and 22°C) and two-factor levels in day length (8 and 16 h) with five replications. Seeds of 184 DH lines, the parental genotypes, and the F1 were sown in two 96 multi-pot trays (Quickpot 96, HerkuPlast Kubern GmbH, Ering, Germany) with a total size of 335 × 515 mm in four duplicates. Single pots had a size of 38 × 38 × 78 mm and were filled with soil (Fruhstorfer Erde type T25, HAWITA Gruppe GmbH, Vechta, Germany) and cultivated for 3 to 4 weeks in the greenhouse until the two- to three-leaf developmental stages (BBCH 12 to 13; Lancashire et al., 1991). Then, the multi-pot trays were transferred to a vernalization chamber adjusted to 4°C–5°C and 8 h cool white light (Schuch Typ 164/12 L96C 82W) for 9 weeks. After vernalization, the plants were transferred to two growth chambers with different temperatures, which were divided with sheets impervious to light to allow treatment with different day lengths. Therefore, the conditions consisted of 4 day length and temperature combinations of 8 h/11°C (SD11), 8 h/22°C (SD22), 16 h/11°C (LD11), and 16 h/22°C (LD22). For testing the effect of day lengths and temperatures, the positions of the genotypes on the multi-pot trays were randomized in each replication and condition. Growth chambers were equipped with Philips MASTER Green Power CG T 400 W, providing light intensities of 110–120 µmol·m^−2^·s^−1^. Plants were watered and fertilized on a regular basis and treated with fungicides and insecticides, when necessary. Days to flowering (DTF) was recorded starting from the day of transfer to the climate chamber. Replications were terminated at day 135. Genotypes that did not flower at day 135 but showed buds were recorded with a value of 150 DTF, and if they did not show buds, they were recorded with a value of 165 DTF. The means over all replications of each condition were used to calculate differences in days to flowering. Differences between DTF under short and long days at the same temperature (SD-LD11 and SD-LD22) and between low and high temperatures under the same day length (11-22LD and 11-22SD) were calculated. A full list of phenotypic data is available in **Supplementary Table S1**.

### 2.3 Statistical analysis

PLABSTAT 3A software (Utz, 2011) was used to calculate the analysis of variance and heritabilities. The ANOVA for day length and temperature experiment was performed using the model for a split–split plot design: $Y_{ijkl}=µ+r_{i}+t_{j}+r_{i}t_{j}+d_{k}+\;t_{j}d_{k}+r_{i}t_{j}d_{k}+g_{l}+g_{l}t_{j}+g_{l}d_{k}+g_{l}t_{j}d_{k}+g_{l}t_{j}d_{k}r_{i}$ , where Y_ijkl_ is the trait value of the genotype l in the day length condition k and the temperature condition j in replication i; μ is the general mean; t_j_ and r_i_ are the effects of temperature j and replication i, respectively; and r_i_t_j_ is the interaction between the ith replication and jth temperature, which is treated as the first stratum error. The effect of the kth day length is d_k_, and t_j_d_k_ is the interaction between the jth temperature and kth day length, r_i_t_j_d_k_ is the second stratum error (interactions between the ith replication, jth temperature, and kth day length; g_l_ is the effect of the lth genotype; g_l_t_j_, g_l_d_k_, and g_l_t_j_d_k_ are the interactions between the lth genotype with the jth temperature and kth day length, while g_l_t_j_d_k_r_i_ is the third stratum error term. The factors genotypes and replications were taken as random. Broad-sense heritabilities were calculated using the formula $H^{2}=\sigma_{g}^{2}\;/\;\left(\sigma_{g}^{2}\;+\;\sigma_{gtde}^{2}\;/T\right)$ , with coefficient T = 20 as the product of all factor levels.

Other statistical analyses were performed in R (R. Core Team, 2019). Figures of the descriptive statistics were created in R using the package ggplot2 (Wickham, 2016; R. Core Team, 2019). A Tukey’s test was used to test significant differences (p ≤ 0.01) between subgroups in figures with a box plot.

### 2.4 QTL analysis

A previously published full marker map consisting of 21,583 markers distributed over 19 linkage groups was used to develop a bin map of 1,883 markers (Valdés et al., 2018). Mean values over the five replications were used in QTL mapping for all traits. QTL mapping was performed using the WinQTL Cartographer software version 2.5 (Wang et al., 2012), and the composite interval mapping (CIM) algorithm was employed with the following specifications: independent logarithm of odds (LOD) significance thresholds (α = 0.05) were estimated for each trait by 1,000 permutation tests. Model 6 was employed; the forward and backward stepwise regression method was used to set cofactors. The genome was scanned at 1-cM intervals, and the window size was set to 10 cM. The 95% confidence interval for each QTL was determined by one LOD drop from the peak position. Additive effects, as well as the percentage of phenotypic variance explained by a QTL, were determined. A positive additive effect of a QTL is an additive effect by the allele of the winter oilseed rape parent Express617. To test epistasis, a multiple-interval mapping method was used. QTLs found in CIM were used as input and the BIC-M0 model with 1-cM walk speed and 10-cM window size. Additive × additive effects were significant with a LOD score of 2.4.

SNP marker sequences were provided by Isobel Parkin (AAFC, Saskatoon, SK, Canada), aligned using the BLAST algorithm against the reference genome sequence of “Express617” (Lee et al., 2020) by use of the Galaxy BLASTn-short algorithm (Cock et al., 2015), and used to create a physical map. Figures of the maps were drawn using MapChart (Voorrips, 2002). Gene annotation was provided by Schilbert et al. (2021).

## 3 Results

### 3.1 Quantifying the effect of day length and temperature on flowering time of fully vernalized plants

According to the analysis of variance, DTF is predominantly influenced by the effect of day length (**Table 1**). The size of the variance components for the effect of day length was almost 20 times that of the temperature and more than two times that of the genotype. The size of the variance components for the temperature × day length interaction was 1.5 times that of the effects of temperature. DTF showed a high broad-sense heritability of *H* ^2^ = 95%. Short-day (SD) conditions (8-h light) delayed the mean DTF in the DH population, as well as for the parents and F1, but also increased the range (**Table 2**).

**Table 1:** Components of variance and broad-sense heritability (*H* ^2^) for days to flowering for the DH population DH4079 × Express617 (n = 184) at two different temperatures T (11°C and 22°C) and two different day length conditions D (8 h and 16 h) after full vernalization treatment.

Source	Degrees of freedom	Components of variance	
Replication (R)	4	36.1	*
Temperature (T)	1	34.9	**
Day length (D)	1	656.9	***
Genotype (G)	183	244.9	***
R × T	4	17.2	**
D × T	1	53.4	***
R × D × T	14	9.3	***
T × G	183	30.1	***
D × G	183	91.2	***
D × T × G	183	12.1	**
R × D × T × G	2,641	236.8	
*H* ^2^ (%)		95	

* p ≤ 0.10, ** p ≤ 0.05, and *** p ≤ 0.01.

**Table 2:** Descriptive statistics for days to flowering of vernalized plants of the DH4079 × Express617 DH population grown under four different conditions, with different temperatures (11°C and 22°C) and different day lengths: short day (SD; 8 h) and long day (LD; 16 h).

Condition	DH lines (n = 184)				Parental genotypes			LSD 5%	*H* ^2^ [%]
	Min	Max	Median	Mean	DH4079	F1	Express617		
LD 11°C	34	89	51	52	38	49	72	9.9	84
LD 22°C	17	105	31	35	17	30	58	13.4	87
SD 11°C	42	153	78	80	52	75	103	15.8	90
SD 22°C	28	165	74	78	33	63	118	29.8	86
Effect of day length differences on DTF									
SD-LD 11°C	−4	66	26	29	15	26	32		
SD-LD 22°C	7	100	40	43	16	34	60		
Effect of temperature differences on DTF									
11-22°C LD	−20	44	18	17	21	19	14		
11-22°C SD	−44	40	5	3	19	12	−15		

Effects of temperature and day length differences on DTF were calculated for each genotype.

LSD 5%, least significant difference; DTF, days to flowering.

**Figure 1:**
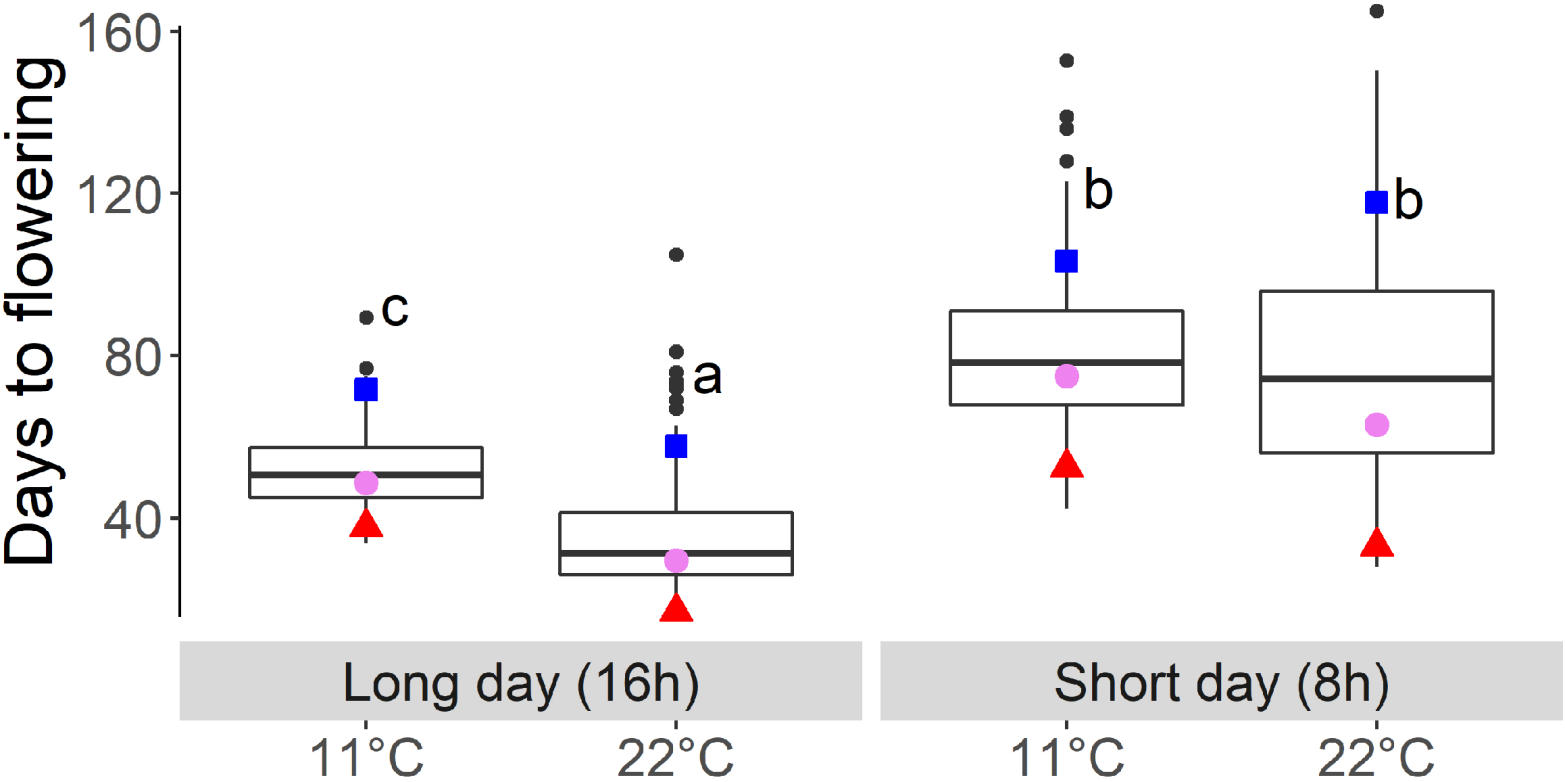
Days to flowering of the fully vernalized DH population growing under different temperatures (11°C and 22°C) and day length (short- and long-day) conditions. Letters indicate significantly different subgroups (p ≤ 0.01) tested with Tukey's test. Winter oilseed rape parent Express617 is indicated with blue square, spring-type parent DH4079 indicated with red triangle, and F1 indicated with violet circle.

The mean of the DH lines showed an earlier flowering time due to higher temperatures under long-day (LD) conditions (16-h light) from 52 days at 11°C to 35 days at 22°C. The range increased under 22°C and SD conditions (**Table 2**, **Figure 1**). Under SD conditions, the means for DTF under the two temperature regimes were no longer significantly different (**Table 2**, **Figure 1**). In all conditions, the winter oilseed rape parent Express617 flowered later than the spring-type parent DH4079, and the F1 showed an intermediate phenotype but had values slightly closer to those of the spring-type parent (**Figure 1**).

The effect of day length differences on DTF in the DH population, calculated by subtracting DTF under LD from DTF under SD, had in a temperature of 22°C a population mean of SD-LD22 = 43 days and in 11°C only SD-LD11 = 29 days (**Table 2**). However, under both temperatures, the range was extensive from −4 up to 100 days of difference in flowering time. In the winter oilseed rape parent Express617, the effects of day length differences were SD-LD11 = 32 days and SD-LD22 = 60 days, while the effects in the spring-type parent DH4079 were lower and similar in both temperatures (SD-LD11 = 15 and SD-LD22 = 16 days).

The effect of temperature differences, calculated by subtracting DTF at 22°C from 11°C at the respective day lengths, showed under LD a population mean of 11-22LD = 17 days and under SD a mean of 11-22SD = 3 days differences in flowering (**Table 2**). The values for this effect of temperature differences on the DH lines ranged between −20 and 44 days under the long-day conditions (11-22LD) and between −44 and 40 days under the short-day conditions (11-22SD). This range showed the ability of warmer temperatures to either accelerate or delay DTF compared to cool temperatures, depending on the genotype and in interaction with the day length conditions. Under LD conditions, the effect of temperature differences on the spring-type parent DH4079 was 11-22LD = 21 days, with an acceleration of flowering through warmer temperatures. In the winter-type parent Express617, the value was lower with 11-22LD = 14 days, and the F1 showed an intermediate phenotype. Under SD, the warmer temperature led to a delayed flowering time in Express617 with 11-22SD = −15 days, while still accelerating in the F1 (11-22SD = 12 days) and DH4079 (11-22SD = 19 days, **Table 2**, **Figure 1**). For DH4079, the effects of temperature differences showed similar values with 11-22LD = 21 days and 11-22SD = 19 days as the effects of day length differences, indicating no interaction between temperature and day length in this parental genotype (**Table 2**).

### 3.2 Identification of major genomic regions with clusters of QTL

**Figure 2:**
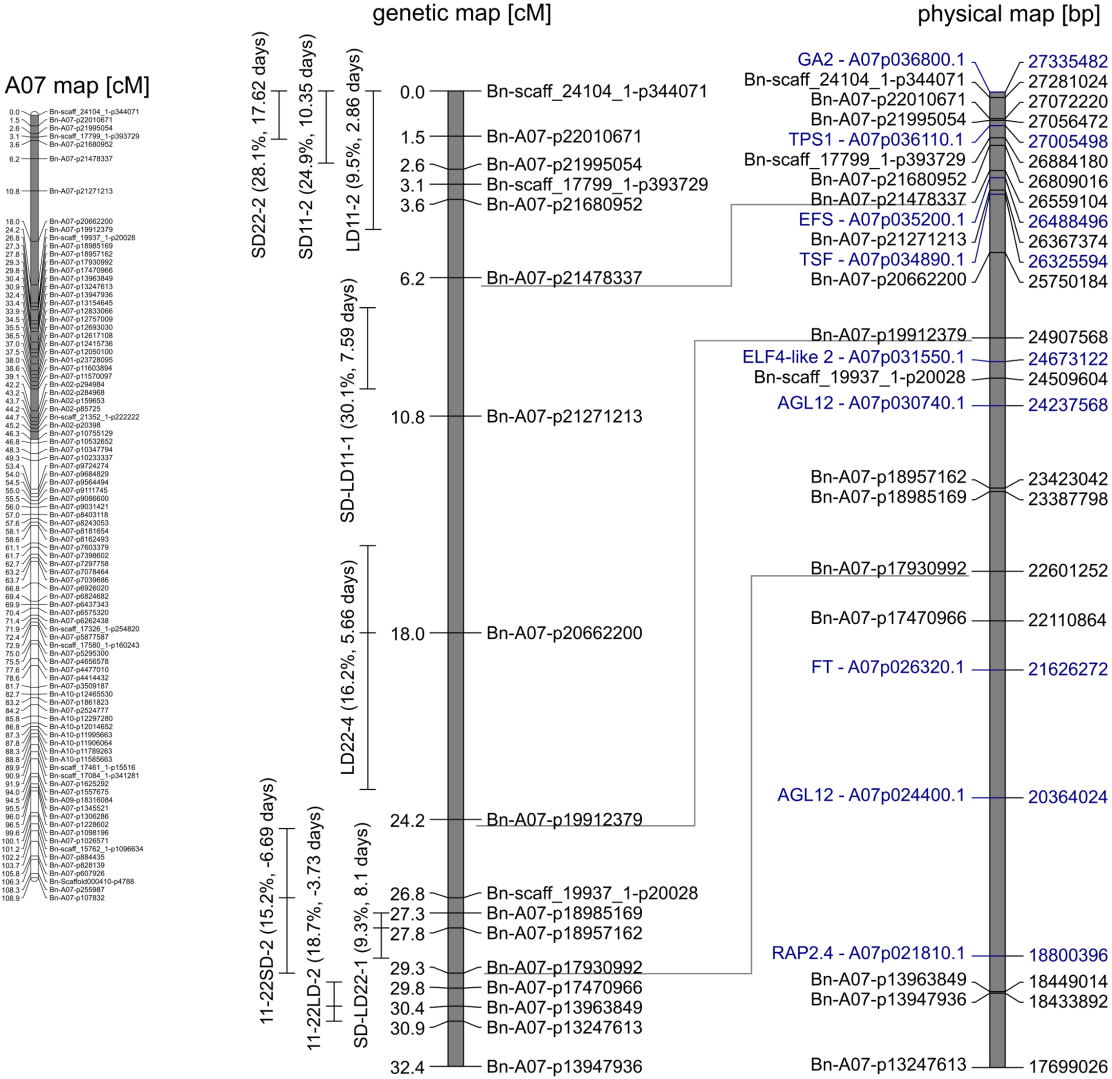
Gray marked in genetic map of A07 (left) is the section enhanced on the right. Genetic (middle) map depicts the quantitative trait loci (QTLs) clustering on A07. QTLs are given with peak and 95% confidence interval. The phenotypic variance explained (in percent) and additive effect (in days) of the QTL are shown in brackets. In the corresponding physical (left) map, candidate genes (blue) and the respective gene ID in the reference genome of “Express617” are shown.

**Figure 3:**
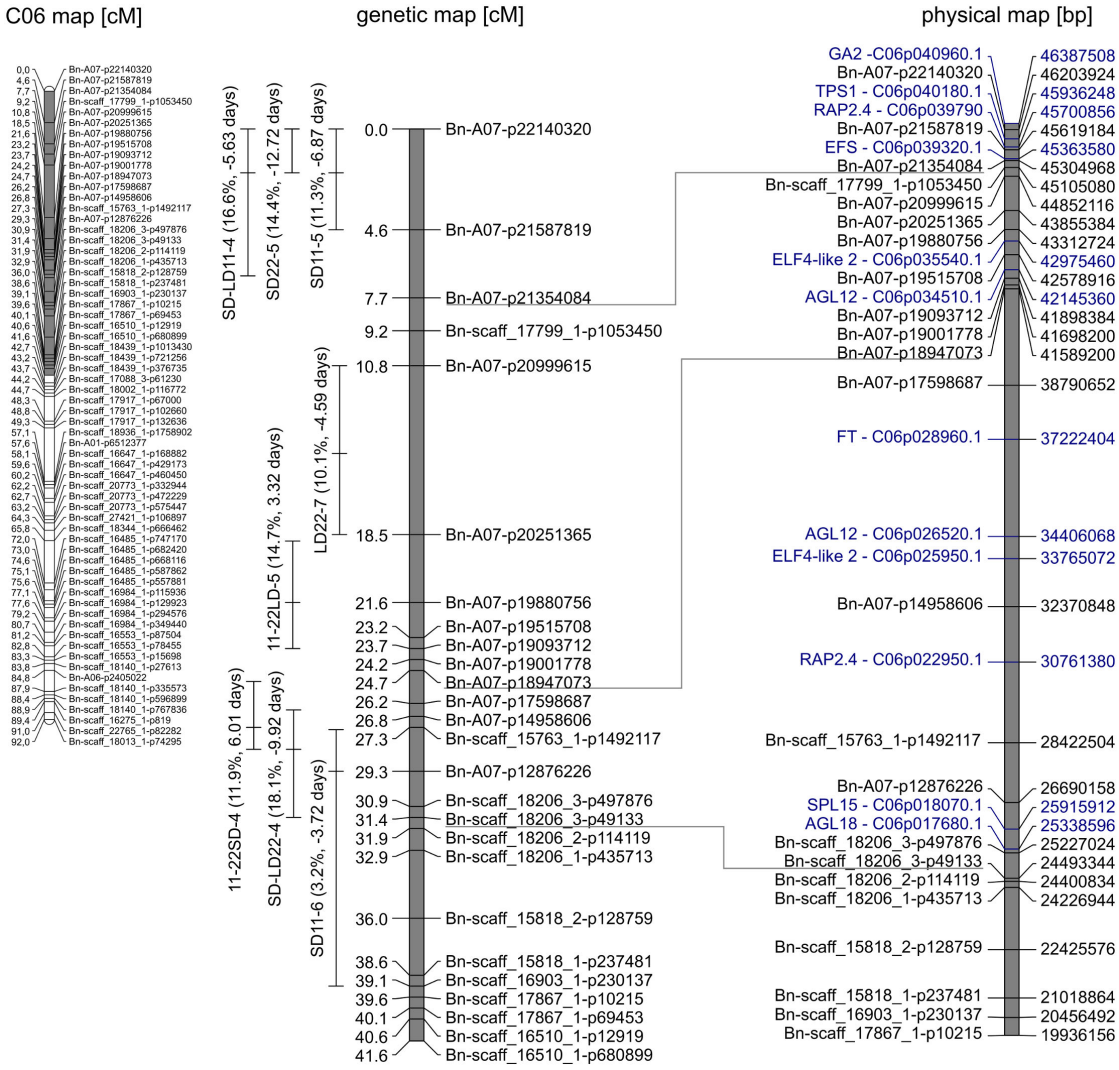
Gray marked in genetic map of C06 (left) is the section enhanced on the right. Genetic (middle) map depicts the quantitative trait loci (QTLs) clustering on C06. QTLs are given with peak and 95% confidence interval. The phenotypic variance explained (in percent) and additive effect (in days) of the QTL are shown in brackets. In the corresponding physical (right) map, candidate genes (blue) and the respective gene ID in the reference genome of “Express617” are shown.

The QTL analysis (**Table 3**) revealed between four and seven QTLs for each trait, which when summed up could explain between TR^2^ = 38.6% and 65.2% of the phenotypic variance of the respective trait (**Table 3**). Most QTLs for days to flowering showed a positive effect, except for QTLs on A05 (LD22-3) and C06 (LD22-7, SD11-5, SD11-6, and SD22-5), indicating that the delay of flowering was caused mainly by Express617 alleles (**Table 3**). Certain regions of the genome showed an accumulation of QTLs. The major QTLs were forming clusters on chromosomes A07 and C06 (**Figures 2**, **3**).

**Table 3:** Quantitative trait loci mapped for DTF under different temperature and day length conditions, the effects of temperature, and the effects of day length in the DH4079 × Express617 population.

QTL_name	Chr.	Position [cM]	CI [cM]^a^			Marker flanking CI left	Marker flanking CI right	LOD	Additive	R^2^ [%]^b^	TR^2^ [%]^c^
Long day at 11°C											
LD11-1	A02	54.4	53.6	–	56	Bn-A01-p16630613	Bn-A02-p8549867	16.0	4.4	23.1	46.4
LD11-2	A07	0.0	0	–	4.6	Bn-scaff_24104_1-p344071	Bn-A07-p21478337	7.4	2.9	9.5	
LD11-3	C02	5.6	3.3	–	7.2	Bn-A02-p1705187	Bn-scaff_15714_1-p3087640	6.3	2.7	7.9	
LD11-4	C02	62.6	58.3	–	65.2	Bn-scaff_23546_1-p103326	Bn-scaff_16449_1-p318325	4.7	2.2	5.9	
Long day at 22°C											
LD22-1	A02	30.3	26.2	–	33.5	Bn-A02-p24449690	Bn-A02-p21930156	10.3	5.2	13.5	64.5
LD22-2	A02	54.4	51.81	–	56.91	Bn-A02-p11713324	Bn-A02-p8057234	4.4	3.8	4.9	
LD22-3	A05	8.7	4.9	–	16.9	Bn-A05-p917769	Bn-A05-p2254100	3.5	−2.7	3.9	
LD22-4	A07	18.0	15.1	–	23.2	Bn-A07-p21271213	Bn-A07-p19912379	11.9	5.7	16.2	
LD22-5	C02	5.6	3.8	–	6.7	Bn-scaff_17752_1-p128342	Bn-scaff_22970_1-p213807	7.2	4.4	9.1	
LD22-6	C02	73.4	71.1	–	77.2	Bn-scaff_15712_2-p104622	Bn-scaff_17109_4-p101748	5.4	3.6	6.7	
LD22-7	C06	14.8	10.8	–	18.5	Bn-A07-p20999615	Bn-A07-p20251365	7.2	−4.6	10.1	
Short day at 11°C											
SD11-1	A02	21.5	21	–	23.6	Bn-A02-p24844291	Bn-A02-p24708197	4.0	4.1	5.0	63.0
SD11-2	A07	0.0	0	–	2.4	Bn-scaff_24104_1-p344071	Bn-A07-p21995054	17.5	10.3	24.9	
SD11-3	C02	3.5	1.5	–	6.7	Bn-A02-p1705187	Bn-scaff_22970_1-p213807	11.0	7.4	14.5	
SD11-4	C02	66.2	65.2	–	69.3	Bn-scaff_16449_1-p318325	Bn-scaff_15712_6-p470292	3.4	3.8	4.2	
SD11-5	C06	2.0	0	–	4.6	Bn-A07-p22140320	Bn-A07-p21587819	8.4	−6.9	11.3	
SD11-6	C06	29.3	27.4	–	39.1	Bn-scaff_15763_1-p1492117	Bn-scaff_16903_1-p230137	2.8	−3.7	3.2	
Short day at 22°C											
SD22-1	A02	52.3	50.3	–	55.9	Bn-A02-p12449263	Bn-A02-p8549867	3.1	5.5	3.4	65.2
SD22-2	A07	0.0	0	–	1.6	Bn-scaff_24104_1-p344071	Bn-A07-p21995054	19.2	17.6	28.1	
SD22-3	C02	11.7	8.2	–	14.2	Bn-scaff_15714_1-p2978071	Bn-scaff_15714_1-p2481342	9.0	10.6	11.7	
SD22-4	C02	73.4	64.21	–	78.01	Bn-scaff_16298_1-p102179	Bn-scaff_17109_1-p1144887	6.1	8.2	7.5	
SD22-5	C06	0.0	0	–	2	Bn-A07-p22140320	Bn-A07-p21587819	11.0	−12.7	14.4	
Effect of day length under 11°C (calculated difference between SD11 and LD11)											
SD-LD11-1	A07	7.2	7.2	–	9.9	Bn-A07-p21478337	Bn-A07-p21271213	18.2	7.6	30.1	60.6
SD-LD11-2	C02	12.3	12.2	–	14.7	Bn-scaff_15714_1-p2978071	Bn-scaff_15714_1-p2481342	7.0	4.2	10.0	
SD-LD11-3	C03	0.5	0	–	1	Bn-scaff_16614_1-p1995086	Bn-scaff_16614_1-p1467715	2.9	2.9	3.9	
SD-LD11-4	C06	2.0	0	–	6.7	Bn-A07-p22140320	Bn-A07-p21354084	10.9	−5.6	16.6	
Effect of day length under 22°C (calculated difference between SD22 and LD22)											
SD-LD22-1	A07	27.8	27.3	–	28.8	Bn-A07-p18985169	Bn-A07-p17930992	6.2	8.1	9.3	38.6
SD-LD22-2	C02	11.7	7.2	–	16.1	Bn-scaff_22970_1-p213807	Bn-A02-p2776634	4.4	5.8	6.7	
SD-LD22-3	C02	80.6	75.41	–	90.31	Bn-scaff_17088_1-p12134	Bn-scaff_17623_1-p119334	3.1	4.5	4.5	
SD-LD22-4	C06	28.3	26.5	–	31.4	Bn-A07-p17598687	Bn-scaff_18206_3-p49133	11.2	−9.9	18.1	
Effect of temperature under LD (calculated difference between LD11 and LD22)											
11-22LD-1	A05	13.8	9.6	–	17.3	Bn-A05-p1347246	Bn-A05-p2254100	4.0	2.1	6.9	51.5
11-22LD-2	A07	30.4	29.6	–	30.9	Bn-A07-p17930992	Bn-A07-p13247613	10.3	−3.7	18.7	
11-22LD-3	C01	40.7	39.1	–	42.6	Bn-scaff_19193_1-p725427	Bn-scaff_17731_1-p166950	2.9	−1.8	4.8	
11-22LD-4	C02	74.4	74.2	–	77.3	Bn-scaff_22144_1-p193415	Bn-scaff_17109_4-p101748	3.7	−2.0	6.2	
11-22LD-5	C06	21.6	18.8	–	23.7	Bn-A07-p20251365	Bn-A07-p19093712	8.3	3.3	14.7	
Effect of temperature under SD (calculated difference between SD11 and SD22)											
11-22SD-1	A05	7.2	4.1	–	8.2	Bn-A05-p761283	Bn-A05-p1335771	4.1	4.3	6.9	39.7
11-22SD-2	A07	26.8	24.5	–	29.3	Bn-A07-p19912379	Bn-A07-p17930992	8.5	−6.7	15.2	
11-22SD-3	C02	76.5	69.81	–	79.51	Bn-scaff_15712_6-p662107	Bn-scaff_17109_1-p859844	3.4	−3.9	5.7	
11-22SD-4	C06	27.3	25.2	–	28.3	Bn-A07-p18947073	Bn-A07-p12876226	6.9	6.0	11.9	

DTF, days to flowering; QTL, quantitative trait locus.

a 95% confidence interval.

b Explained phenotypic variance of the QTL.

c Phenotypic variance explained by all QTLs of the trait.

#### 3.2.1 QTLs on chromosome A07

On the linkage group A07, two QTL clusters were identified (**Figure 2**). At the beginning of the genetic map between 0 and 9.9 cM, four QTLs were located, three of which are related to flowering under short days (SD11-2, SD22-2, SD-LD11-1, and LD11-2). The major QTLs of both traits regarding DTF under SD conditions collocated here; QTL SD11-2 at 0 cM explained R^2^ = 24.9% of the variance with an additive effect of 10.3 days, and QTL SD22-2 at 0 cM explained R^2^ = 28.1% with an additive effect of a = 17.6 days. The second-largest QTL LD11-2 for the trait DTF under 11°C and LD was positioned at 0 cM with an explained variance of R^2^ = 9.5% and an additive effect of a = 2.9 days. Since the peaks for those three QTLs were located at 0 cM, the real QTLs may be located outside of the genetic map. The major QTL for the effect of day length differences at 11°C, SD-LD11-1, was located on A07 at 7.2 cM with an explained variance of R^2^ = 30.1% and an additive effect of a = 7.6 days. For all four QTLs, the Express617 allele delayed flowering. Between 24.5 and 30.9 cM on the same chromosome A07, three QTLs formed a second cluster; two major QTLs for the temperature differences were mapped here, even though the confidence intervals did not overlap (**Figure 2**), and one QTL for day length differences at 22°C was mapped. For temperature differences under LD, the largest QTL 11-22LD-2 at 30.4 cM explained a phenotypic variance of R^2^ = 18.7% and an additive effect of a = −3.7 days. For temperature differences under SD, the largest QTL 11-22SD-2 mapped at 26.8 cM and explained R^2^ = 15.2% of the phenotypic variance with an additive effect of a = −6.7 days. Their additive effects were negative, meaning that the Express617 allele made this effect smaller by either delaying DTF at 22°C or accelerating DTF under 11°C. The second-largest QTL for day length differences at 22°C (SD-LD22-1) was located on A07 at 27.8 cM, falling into the confidence interval of 11-22SD-2 (**Figure 2**), and explained R^2^ = 9.3% of the phenotypic variance (a = 8.1 days).

#### 3.2.2 QTLs on chromosome C06

At the beginning of C06 from 0 to 6.7 cM, a QTL cluster for DTF in SD was located (**Figure 3**). The second-largest QTL for DTF in warm SD conditions, SD22-5, was located on C06 at 0 cM (R^2^ = 14.4%, a = −12.7 days). It had overlapping confidence intervals with the third largest QTL for DTF in cool SD conditions, SD11-5 (R^2^ = 11.3%, a = −6.9 days), and the second-largest QTL for the effect of day length difference under 11°C, SD-LD11-4 (R^2^ = 16.6%, a = −5.6 days), which were both collocated on C06 at 2 cM.

On the same chromosome C06 between 18.8 and 39.1 cM, a QTL cluster with four QTLs regarding the reaction of DTF to day length and temperature was located. The effects of temperature difference under SD and LD were both mapped with their second-largest QTL in this cluster, although they showed no overlapping confidence intervals. QTL 11-22LD-5 on C06 at 21.6 cM explained R^2^ = 14.7% of the variance with a positive additive effect of a = 3.3 days. QTL 11-22SD-4 on C06 at 27.3 cM explained R^2^ = 11.9% of the variance with a positive additive effect of a = 6.0 days. The effect of day length differences at 22°C had its major QTL SD-LD22-4 on C06 at 28.3 cM with R^2^ = 18.1% explained variance and an additive effect of a = −9.9 days, meaning the DH4079 allele at this position delayed flowering under SD. The DTF under SD and 11°C mapped with the minor QTL SD11-6 at 29.3 cM and an explained variance of R^2^ = 3.2% and a = −3.7 days. The three QTLs 11-22SD-4, SD-LD22-4, and SD11-6 had overlapping confidence intervals. Interestingly, the Express617 allele accelerated flowering under short days and warm temperatures in both clusters, contrary to the cluster on A07.

#### 3.2.3 Epistatic effects between QTLs on A07 and C06

**Figure 4:**
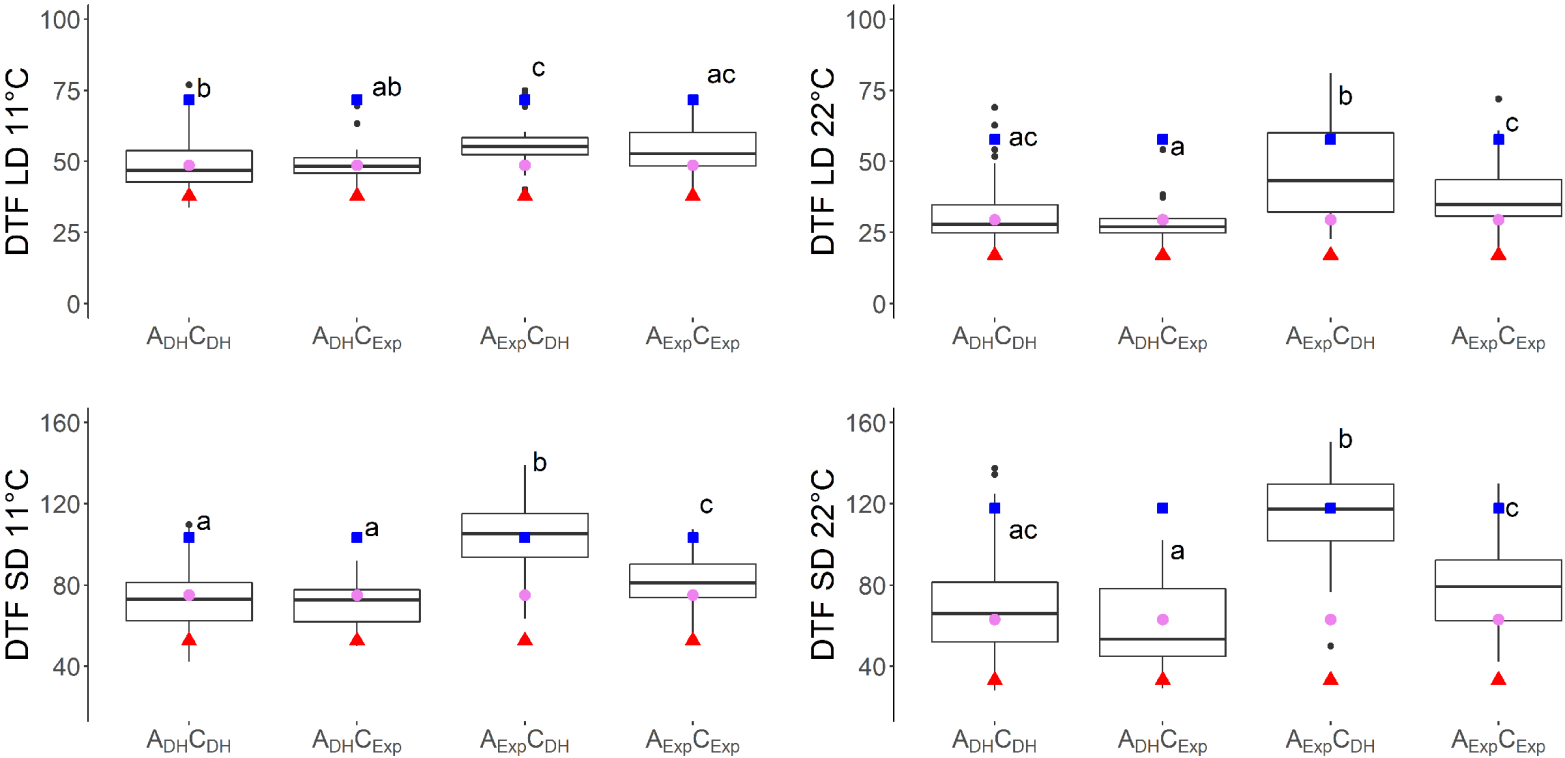
Days to flowering (DTF) in different temperatures (11°C and 22°C) and day length [short day (SD) and long day (LD)] conditions of vernalized DH population divided by alleles of two SNP markers: Bn-A07-p21478337 on A07 at 6.2 cM, indicated by A, and Bn-A07-p21354084 on C06 at 7.7 cM, indicated by C. Subscript “DH” indicates parental DH4079 allele, and subscript “Exp” indicates parental Express617 allele. Letters indicate significantly different subgroups (p ≤ 0.01) tested with Tukey's test within conditions. Phenotypic values of Express617 (blue square), F1 (pink circle), and DH4079 (red triangle) are given.

**Figure 5:**
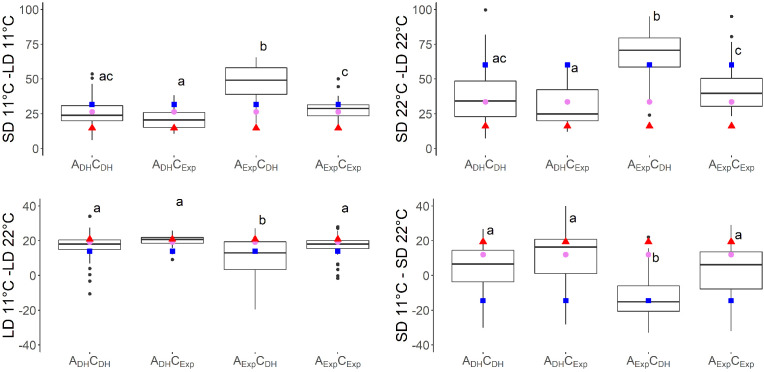
Effects of temperature and day length calculated by subtracting days to flowering (DTF) in different temperatures (11°C minus 22°C) and day length [short day (SD) minus long day (LD)] conditions of vernalized DH population divided by alleles of two SNP markers: Bn-A07-p21478337 on A07 at 6.2 cM, indicated by A, and Bn-A07-p21354084 on C06 at 7.7 cM, indicated by C. Subscript “DH” indicates parental DH4079 allele, and subscript “Exp” indicates parental Express617 allele. Letters indicate significantly different subgroups (p ≤ 0.01) tested with Tukey's test within conditions. Phenotypic values of Express617 (blue square), F1 (pink circle), and DH4079 (red triangle) are given.

Seven traits showed a QTL on both A07 and C06. For six of these traits, additive × additive epistatic effects were found between QTLs on chromosomes A07 and C06. The epistatic effect between QTL SD11-2 on A07 and QTL SD11-5 on C06 with an effect of a × a = −7.0 was the strongest epistatic effect in this study (**Table 4**). When grouping the DH population by the alleles of two markers located near the short day-sensitive clusters, one on A07 (Bn-A07-p21478337, 6.2 cM) and one on C06 (Bn-A07-p21354084, 7.7 cM), the epistatic effect can be observed in the phenotype (**Figures 4**, **5**). Tukey’s test between the four haplotype groups showed no significant difference between the two groups that shared the DH4079 allele on A07 (A_DH_C_DH_ and A_DH_C_Exp_) under any condition. Therefore, the DH allele on A07 masked the allelic effect on C06. Except for cool long-day conditions at 11°C, the allele combination A_Exp_C_DH_ resulted in a late flowering group of genotypes.

**Table 4:** Epistatic effects between QTLs (see **Table 3**) for days to flowering (DTF) under different day length and temperature conditions, as well as for the effect of day length and temperature in the DH4079 × Express617 population.

1st QTL	Chr.	Pos. [cM]	Interaction with	“2nd QTL”	Chr.	Pos. [cM]	Additive × additive effect
LD22-2	A02	54.4	×	LD22-4	A07	18.0	1.8
LD22-1	A02	30.3	×	LD22-5	C02	5.6	2.2
LD11-1	A02	54.4	×	LD11-4	C02	62.6	0.5
LD22-1	A02	30.3	×	LD22-7	C06	14.8	−3.1
LD22-4	A07	18.0	×	LD22-5	C02	5.6	1.7
11-22LD-2	A07	30.4	×	11-22LD-4	C02	74.4	−2.3
**SD22-2**	**A07**	**0.0**	×	**SD22-5**	**C06**	**0.0**	−5.2
**SD11-2**	**A07**	**0.0**	×	**SD11-5**	**C06**	**2.0**	−7.0
**SD-LD11-1**	**A07**	**7.2**	×	**SD-LD11-4**	**C06**	**2.0**	−3.9
**LD22-4**	**A07**	**18.0**	×	**LD22-7**	**C06**	**14.8**	−2.1
**SD-LD22-1**	**A07**	**27.8**	×	**SD-LD22-4**	**C06**	**28.3**	−4.5
**11-22LD-2**	**A07**	**30.4**	×	**11-22LD-5**	**C06**	**21.6**	1.6
SD11-3	C02	3.5	×	SD11-6	C06	29.3	−2.1
LD22-5	C02	5.6	×	LD22-7	C06	14.8	−2.3
11-22LD-4	C02	74.4	×	11-22LD-5	C06	21.6	1.8

QTL, quantitative trait locus; LD, long day; SD, short day.Epistatic interactions between QTL cluster on A07 and C06 marked in bold.

### 3.3 Presentation of known flowering time genes in the QTL regions on A07 and C06

The cluster for DTF under short days at the beginning of the genetic map of A07 consisted of three QTLs (SD11-2, SD22-2, and LD11-2). Since the peaks for those three QTLs were located at 0 cM, the real QTLs may be located outside of the genetic map. Two possible candidate genes were located in this genomic region: *GA REQUIRING 2* (*GA2*) was located outside of the genetic map, and *TREHALOSE-6-PHOSPHATE SYNTHASE 1* (*TPS1*) was located between two markers in the confidence interval of QTL LD11-2 (**Figure 2**). As a possible candidate gene, *EARLY FLOWERING IN SHORT DAYS* (*EFS*) was identified for the QTL for the effect of day length differences at 11°C, SD-LD11-1, at 7.2 cM (**Figure 2**).

The second QTL cluster formed between 24.5 and 30.9 cM on chromosome A07. Here, two major QTLs for the temperature differences were mapped (11-22SD-2 and 11-22LD-2), as well as one QTL for day length differences at 22°C (SD-LD22-1). No known flowering time gene could be found for the confidence interval of SD-LD22-1. With regard to the QTL for temperature difference, the confidence interval of QTL 11-22SD-2 covered a genomic region with *AGAMOUS-LIKE 12* (*AGL12*) and *EARLY FLOWERING4-like 2* (*ELF4-like2*) homologs. The confidence interval of QTL 11-22LD-2 included copies of *FLOWERING LOCUS T* (*FT*), *AGL12*, and *RELATED TO AP2 4* (*RAP2.4*) as possible candidate genes (**Figure 2**).

At the beginning of C06, the QTL cluster for DTF in SD was located from 0 to 6.7 cM (**Figure 3**). The peak of SD22-5 and the confidence intervals of all three reached the end of the genetic map, which means that genes located outside the genetic map should be considered; therefore, *GA2* is another possible candidate gene (**Figure 3**) similar to the cluster on A07. Homologs of *TPS1* and *RAP2.4* were identified as candidate genes for QTLs SD22-5, SD11-5, and SD-LD11-4; *EFS* was identified as an additional possible candidate gene for SD-LD11-4 (**Figure 3**). On chromosome C06 between 18.8 and 39.1 cM, a QTL cluster with four QTLs regarding the reaction of DTF to day length and temperature was located. For QTLs 11-22SD-4 and SD-LD22-4, *FT*, *AGL12*, *ELF4-like2*, and *RAP2.4* were identified as possible candidate genes. The genes *SPL15* and *AGL18* are possible candidate genes for SD-LD22-4 and SD11-6.

The physical maps of A07 and C06 show many homologs of the same genes in between them, and QTLs of the same trait are often located near homologous genes on A07 and C06 (**Figures 2**, **3**). According to Chalhoub et al. (2014), these regions are homoeologous in the reference genome Darmor-*bzh*. Our results indicate that the homology between the chromosomes is also true for the used reference genome Express617 (Lee et al., 2020) and our population.

## 4 Discussion

### 4.1 Short days delay flowering after vernalization

The aim of this study was to close a research gap in quantifying the effect of temperature and day length after vernalization on DTF, especially in material with a genetic background in both spring- and winter-type oilseed rape. Plants were vernalized for 9 weeks and grown in two different day length conditions (8 and 16 h) as well as two different temperature conditions (11°C and 22°C). With regard to day length, it was confirmed that short-day conditions delay flowering time. The genetic analysis showed that QTLs of the same trait mapped on homoeologous regions on A07 and C06, which, therefore, resulted in similar candidate genes and epistatic effects.

Previous studies in *Arabidopsis* and *Brassica* have shown that these long-day plants show a late flowering genotype under SD conditions. In *Brassica* species, a change of day length between 12 and 14 h showed the biggest response (King and Kondra, 1986; Nanda et al., 1996). Later flowering genotypes showed stronger responses to photoperiod than early flowering genotypes (King and Kondra, 1986; Robertson et al., 2002). The DH population of this study also showed a strong delay of DTF under SD conditions after the vernalization requirement was fulfilled, even to the point where some genotypes did not even start flowering at the end of the experiment (**Figure 1**). However, genetic variation in the amount of delay was observed as shown by the calculation of the effects of day length (SD-LD11°C and SD-LD22°C) and the range of DTF (**Table 2**, **Figures 1**, **5**). The delay of flowering under short days was observed in both parental genotypes (**Table 2**; **Figure 1**). The QTL analysis revealed two important genomic regions, where several QTLs for DTF under SD conditions and the effects of day length differences formed clusters. The clusters were located on homoeologous regions on A07 and C06, which means that they were orthologous in the two ancestral species of *B. napus*: *B. rapa* and *B. oleracea* (Chalhoub et al., 2014). The cluster on chromosome A07 was located between 0 and 9.9 cM, where QTLs for DTF under short day (SD22-2 and SD11-2) collocated and a QTL for the effect of day length differences at 11°C SD-LD11-1 was located nearby (**Figure 2**). The cluster at the beginning of the genetic map of C06 was located between 0 and 6.7 cM, where the confidence interval of QTL SD22-5 overlapped with that of QTLs SD11-5 and SD-LD11-4 (**Figure 3**). However, the direction of the additive effect was different between the clusters on A07 and C06. For the QTL on A07, the winter oilseed rape Express617 allele delayed flowering in short-day conditions. In contrast to that, the additive effect was negative for the QTL on C06 with the spring-type DH4079 allele delaying flowering in short days. Additionally, epistatic effects were recorded between the respective QTLs (**Table 4**). The DH alleles on A07 masked the allelic effect on C06, as the group with the A_DH_C_DH_ haplotype and the group with the A_DH_C_Exp_ haplotype showed no significant difference in their means according to Tukey’s test (**Figures 4**, **5**). The allele combination A_Exp_C_DH_ resulted in the largest delay in flowering under short days and also warm long-day conditions (**Figure 4**), as can be seen especially in the effect of day length differences, where this allele combination was always significantly different from the others (**Figure 5**). In both homologous genomic regions, copies of the flowering time candidate genes *GA2*, *EFS*, and *TPS1* were located. In *A. thaliana*, TPS1 is the protein responsible for the synthesis of trehalose-6-phosphate (T6P), a sugar signal. TPS1 is necessary for the expression of *FT* and other flowering-inducing genes (Wahl et al., 2013). However, there is no current knowledge about the influence of *TPS1* on flowering through the day length pathways. The protein GA2 is part of the gibberellin synthesis (Berardini et al., 2015). Gibberellin (GA) is a phytohormone that activates flowering but is also involved in many other developmental and stress pathways. Variations in the gene *GA2* would, therefore, influence more phenotypic traits than flowering time. EFS is known in *Arabidopsis* as an *FLC* activator (recruited by the *PAF1*-like complex), meaning its activity delays flowering (Kim et al., 2005). Kim et al. (2005) showed that a mutation in *EFS* accelerates flowering time under short days more than *fri* or *flc* mutations with an active *EFS*. Thus, there is an *FLC*-independent effect of *EFS* on flowering time under short days, whose mechanism is not yet known.

Previous QTL studies in *B. napus* did not present photosensitive QTLs on A07 and C06 but on several other parts of the genome on chromosomes A02, A03, A10, C03, C04, C05, and C09 (Robert et al., 1998; Axelsson et al., 2001; Cai et al., 2008; Luo et al., 2014; Rahman et al., 2018). Therefore, the homoeologous regions on A07 and C06 are novel discoveries for day length-dependent flowering time regulation. This is not surprising considering the complexity of the flowering time regulation. Furthermore, DH4079 contributed an allele on A07, which masked the effect of the C06 alleles (**Table 3**; **Figure 4**). The C06 DH4079 allele could only delay flowering in the absence of the A07 DH4079 allele (**Figure 4**); hence, the C06 DH4079 allele is masked in the parent. In conclusion, epistatic effects should not be ignored, especially in a plant with a complex genome like *B. napus*, where several homolog copies of a gene exist. Further research is needed to reveal the complexity of flowering time regulation found in *B. napus* and in these two homolog regions.

### 4.2 The effect of temperature on flowering time is dependent on day length and genotype

We calculated the effect of temperature on DTF by subtracting DTF at 22°C from DTF at 11°C (11°–22°C); a positive value stands for earlier flowering at warmer temperatures and a negative number for later flowering. Surprisingly, higher temperatures could either delay or accelerate flowering, depending on the genotype (**Table 2**). While most plant species react with earlier flowering to higher temperatures due to warmer temperatures, it has been shown that high temperatures can also delay flowering (Fitter and Fitter, 2002; Jiang, 2023), something also observed in *B. napus* (Abelenda et al., 2023).

The ANOVA showed that the effect of temperature on DTF was much smaller than the effect of the interaction between temperature and day length (**Table 1**). In the phenotypic analysis, DH4079 as a Swedish spring type showed a reaction to temperature that was independent of day length and vice versa (**Table 2**), so when sown in spring, the cultivar can react to warm temperatures without a negative interaction with day length. In contrast, German winter oilseed rape Express617 reacted differently to warmer temperatures depending on the day length; under LD, the flowering was 14 days earlier at 22°C, but under SD warmer temperatures, the flowering was delayed by 15 days. This is the first study to show how important it is to research the combined effect of day length and temperature after vernalization in winter oilseed rape. It should be noted, that in this experiment, the temperatures were constant and did not change between night and day. Other conditions may have led to different results since thermosensing is discussed to vary between day and night (Xiao et al., 2013; Hayes et al., 2021).

We found that in our QTL analysis, homoeologous regions on the linkage groups C06 and A07 responsible for the regulation of flowering time with QTL clusters for traits related to flowering depended on temperature and day length (**Table 3**, **Figures 2**, **3**). The cluster on A07 between 24.5 cM and 30.9 cM was comprised of QTLs 11-22LD-2, 11-22SD-2, and SD-LD22-1 (**Figure 2**). The cluster on C06 between 18.8 and 39.1 cM was comprised of QTLs 11-22LD-4, 11-22SD-5, SD-LD22-4, and SD11-6 (**Figure 3**). On A07, it was the Express617 allele and, on C06, the DH4079 allele that delayed DTF at 22°C or under short days. The two homoeologous clusters showed epistatic effects between QTLs 11-22LD2 and 11-22LD-5 (a × a = 1.6), SD-LD22-1 and SD-LD22-4 (a × a = −4.5), and LD22-4 and LD22-7 (a × a = −2.1). QTLs 11-22SD-2 and 11-22SD-4 showed no epistasis. In each of those genomic regions, homologous copies of the flowering regulator genes *ELF4-LIKE 2*, *AGL12*, *RAP2.4*, and *FT* were located. No details about the function of *ELF4-LIKE 2* are known yet. In *Arabidopsis*, *ELF4-LIKE 2* could not rescue *elf4* mutants (Lin et al., 2019), which does not exclude another function in flowering time regulation. In *Arabidopsis*, *AGL12* has been shown to positively regulate flowering specifically in the apical meristem due to long-day conditions or independently due to higher temperatures (Tapia-López et al., 2008; Rodríguez-Bolaños et al., 2023). However, vernalization has been reported to negate the influence of *AGL12* (Tapia-López et al., 2008), and in our experiment, vernalization was applied. Transcription factor RAP2.4 is involved in many plant developmental processes. Its transcription is induced by abiotic stress, including heat stress, and is reduced by light, and overexpression has been shown to result in earlier flowering time in *Arabidopsis* (Lin et al., 2008). A temperature-dependent activity of the FT protein was shown, as low temperatures reduce the function of FT in *Arabidopsis* (Teper-Bamnolker and Samach, 2005). Under warm short-day conditions, PIF4 is positively regulating *FT* by binding to the promotor and activating transcription (Kumar et al., 2012). Changes in the promotor region of *FT* could cause genotype-specific differences in the regulation of flowering time by other transcription factors. For *B. napus*, differences in *FT* expression have been shown in spring-type cultivars between 21°C and 28°C (Abelenda et al., 2023) for *FT* homologs A02, A07, and C06. Ghanbari (2016) found *FT* on C06 as the candidate gene for a flowering time QTL in an autumn sown field trial of the DH population of Sansibar × Oase, two winter oilseed rape genotypes, thus influencing flowering time in spring after full vernalization. It can be concluded that there is a genotype-specific interaction between temperature and day length. When sown in the field in autumn, winter oilseed rape should not induce flowering on warm winter days, while spring types may induce flowering earlier if warm temperatures permit it. Due to the different regulations of *FT* through temperature under short days, *FT* is a viable candidate gene, already supported by other studies on chromosomes A07 and C06 (Abelenda et al., 2023; Ghanbari, 2016).

### 4.3 Outlook: more research on the effect of changing climate conditions in spring on winter rapeseed is needed

In conclusion, day length had an immense influence on flowering time in the DH4079 × Express617 population, and novel QTL regions were discovered on chromosomes A07 and C06. The gene *EFS*, which represses flowering under short days, was identified as a viable candidate gene. The influence of temperature × day length interactions on flowering time after vernalization is less studied for rapeseed, although with pending climate change, this may become an issue when warm spring temperatures shift to earlier months when the days are shorter. We found that the effects for temperature and day length interactions are greater than just the temperature effect and suggest that these two important abiotic factors should not be studied independently. The effect of temperature under short days is also genotype-specific, and the combination of higher temperatures and short-day conditions can either delay or accelerate flowering time.

Both parental genotypes had alleles, which suppressed flowering under short days and warm temperatures, but on different loci. On C06, the alleles derived from the spring-type parent DH4079 were responsible for this effect, while on A07, the alleles from the winter-type parent Express617 were responsible. In the presence of the DH4079 allele on A07, the effects of the alleles on C06 were masked, and the delay in flowering time through short-day conditions was not expressed. The QTLs on C06 and A07 were located in homoeologous regions and resulted consequently in the same candidate genes. This genetic diversity is a valuable basis for breeding *B. napus* to counter the environmental effects of climate change.

## Data Availability

The original contributions presented in the study are included in the article/**Supplementary Material**. Further inquiries can be directed to the corresponding author.

## Supplementary material

Supplementary File S2 Chat log with Chat-AI to improve the abstract and title.
